# Association between admission baseline blood potassium levels and all-cause mortality in patients with acute kidney injury combined with sepsis: A retrospective cohort study

**DOI:** 10.1371/journal.pone.0309764

**Published:** 2024-11-20

**Authors:** Yifan Guo, Yue Qiu, Taiqi Xue, Pu Yan, Wenjing Zhao, Mengdi Wang, Cheng Liu, Ning Zhang

**Affiliations:** 1 Department of Endocrinology and Nephropathy, Wangjing Hospital of China Academy of Chinese Medical Sciences, Beijing, China; 2 Graduate School, Beijing University of Chinese Medicine, Beijing, China; 3 Department of Endocrinology, Miyun Hospital District, The Third Affiliated Hospital of Beijing University of Chinese Medicine, Beijing, China; 4 Department of Nephrology, Beijing Hospital of Traditional Chinese Medicine, Capital Medical University, Beijing, China; 5 Department of Human Anatomy, Program for Cancer and Cell Biology, Histology and Embryology, School of Basic Medical Sciences, Peking University Health Science Center, Beijing, China; Isfahan Cardiovascular Research Center, ISLAMIC REPUBLIC OF IRAN

## Abstract

**Introduction:**

Imbalances in blood potassium (K) homeostasis is a significant contributor to the emergence of severe complications, especially among critically ill patients. Hypokalemia and hyperkalemia are both associated with an increased risk of adverse events. However, it is not known about the impact of blood K levels on risk of intensive care units (ICU) mortality for Acute kidney injury (AKI) combined with sepsis patients. This study aimed to explore the relationship between admission blood K levels and ICU 30-day mortality in patients with AKI combined with sepsis.

**Methods:**

We selected patients diagnosed with AKI and sepsis on their first ICU admission from the Medical Information Mart for Intensive Care IV (MIMIC-IV) database. The first blood K levels within 24 hours of admission were categorized into three groups according to tertiles (T1 < 3.9 mmol/L, 3.9 ≤ T2 < 4.5 mmol/L, and T3 ≥ 4.5 mmol/L), with T2 serving as the reference. We examined the association between blood K levels and ICU 30-day mortality using accelerated failure time (AFT) models and survival analysis.

**Results:**

A total of 8,242 ICU patients with AKI combined with sepsis were included. In multivariate AFT models, each 1 mmol/L increase in blood K levels was associated with a 13% increase in the risk of ICU 30-day mortality (*p* < 0.001, 95% confidence interval (CI): 1.06–1.20). Extended multivariable AFT models showed that, compared to the middle category, patients with high blood K levels (≥ 4.5 mmol/L) were associated with all-cause mortality (*p* = 0.002, adjusted hazard ratio (HR) = 1.22, 95% CI: 1.08–1.38), whereas those with low blood K levels (< 3.9 mmol/L) showed no significant difference (*p* = 0.385, adjusted HR = 1.06, 95% CI: 0.93–1.21). Kaplan-Meier curves indicated that patients with high blood K levels had higher mortality, and those with middle blood potassium levels (3.9 ≤ K < 4.5 mmol/L) had the lowest mortality.

**Conclusion:**

The admission baseline blood K levels were significantly associated with ICU 30-day mortality in intensive care patients suffering from AKI in conjunction with sepsis. Therefore, immediate and careful correction of blood potassium imbalances may prove to be a crucial approach in improving outcomes for these patients.

## Introduction

Acute kidney injury (AKI) represents a condition characterized by a rapid decrease in renal function over a short period caused by various factors [1]. It affects more than 50% of intensive care unit (ICU) patients and is associated with a significant increase in mortality rate [2,3]. Sepsis, a critical condition resulting from the body’s overwhelming inflammatory response to infection, is the leading cause of AKI among ICU patients [4]. It is reported that the annual direct medical costs associated with AKI are approximately $10 billion [5], while the annual medical expenses for sepsis are even more substantial, amounting to approximately $20 billion [6]. Both conditions contribute to a substantial economic burden on the United States healthcare system [7]. The interplay between AKI and sepsis is intricate; AKI can exacerbate organ dysfunction and accelerate sepsis progression, while sepsis can lead to multiple organ failures, with AKI being a prominent manifestation [8]. Notably, the combination of AKI and sepsis frequently occurs in ICU, and is significantly associated with increased rates of complications and mortality [9,10]. Therefore, the investigation and identification of relevant clinical predictors are essential for improving the prognosis of these patients.

Potassium ion (K+), one of the cations widely located in intracellular, plays a crucial role in the maintenance of cell membrane potential, pH stability, metabolism and energy homeostasis, as well as the regulation of many cellular functions [11,12]. Under physiological conditions, blood K levels are meticulously regulated to remain within 3.5 mmol/L to 5.5 mmol/L mainly through glomerular filtration, tubular reabsorption and secretion, and transfer between intracellular and extracellular fluids [13,14]. Studies have shown that imbalances in potassium homeostasis (including hyperkalemia and hypokalemia) are significantly associated with worse prognosis in a variety of diseases, including cardiac arrhythmia, acute myocardial infarction, heart failure, and chronic kidney disease [14–17]. It is worth noting that the kidney plays a core role in regulating potassium homeostasis in the body, and hyperkalemia has been shown to be one of the major electrolyte disorders in patients with AKI [18]. Hyperkalemia can not only cause cardiac conduction abnormalities in patients with AKI but can also lead to cardiac arrest in severe cases [19,20]. Moreover, it is also associated with the deterioration of renal function, increased mortality rates, and prolonged recovery times [21]. Despite existing studies reporting a significant correlation between elevated blood K levels and increased risk of mortality in patients with AKI, there is still no consistent standard regarding the definition of higher blood K levels in these studies [22–24]. Additionally, electrolyte abnormalities, particularly hyperkalemia, are commonly observed in patients with sepsis [25,26]. On the one hand, the inflammatory response and tissue injury associated with sepsis can affect the distribution of potassium ions and the kidney’s ability to excrete potassium, leading to hyperkalemia [27,28]. On the other hand, hyperkalemia can further exacerbate the hemodynamic instability present in sepsis, thereby reducing tissue perfusion and increasing the mortality rate among patients [29]. However, the short-term prognosis of different blood K levels in patients with AKI combined with sepsis is unknown. Therefore, the purpose of this study was to investigate the correlation between admission baseline blood K levels and ICU 30-day mortality in patients with AKI and sepsis.

## Methods

### Data sources

This was a retrospective cohort study based on the Medical Information Mart for Intensive Care-IV (MIMIC-IV v2.1) database (https://mimic.mit.edu) from 2008 to 2019, which contains the medical records of 523,740 in-patients who admitted to the Beth Israel Deaconess Medical Center’s ICUs in Boston, MA [30]. The database is accessible to individuals who have completed the Collaborative Institutional Training Initiative (CITI) course and related examinations (Certification number 9018458 for Dr. Guo). Users can survey and extract information including demographic characteristics, vital signs, laboratory tests, diagnostic information, medication usage and other related information of each patient. Since the public database is anonymous, the requirements for informed consent and ethical approval were waived. This investigation adhered to the tenets of the Declaration of Helsinki and conformed to the Strengthening the Reporting of Observational Studies in Epidemiology (STROBE) guidelines [31].

### Study population

According to the Kidney Disease: Improving Global Outcomes (KDIGO) guidelines [32], AKI diagnosis criteria include: serum Cr ≥ 1.5 times baseline or ≥ 0.3 mg/dL increase within 48 hours, or urine volume < 0.5 mL/kg/h for ≥ 6 hours. The stages of AKI are defined by the KDIGO guidelines as follows [32]: i) Stage 1: Increase in serum Cr to 1.5–1.9 times baseline or an increase of ≥ 0.3 mg/dL, or urine output < 0.5 mL/kg/h for 6–12 hours. ii) Stage 2: Increase in serum Cr to 2.0–2.9 times baseline, or urine output < 0.5 mL/kg/h for ≥ 12 hours. iii) Stage 3: Increase in serum Cr to 3.0 times baseline; increase in serum creatinine to ≥ 4.0 mg/dL; initiation of renal replacement therapy; in patients < 18 years, decrease in eGFR to 35 mL/min/1.73 m^2^; or urine output < 0.3 mL/kg/h for ≥ 24 hours or anuria for ≥ 12 hours. According to the Third International Consensus Definitions for Sepsis and Septic Shock (Sepsis-3) criteria, sepsis is defined as sequential organ failure assessment (SOFA) ≥ 2 and the presence of infection or suspected infection [33]. All participants should meet the following criteria for inclusion: (a) patients admitted to ICU for the first time; (b) diagnosed both AKI and sepsis within 48 hours of admission; (c) adult patients (≥18 years). Exclusion criteria included: (a) stayed <24 h in the ICU; (b) missing data on blood potassium and other covariates. Eventually, 8,242 patients with AKI combined with sepsis were included in the study.

### Blood potassium measures

The admission baseline blood potassium levels were defined as the first blood K measurement within 24 hours of admission to the ICU. We categorized blood K levels into three groups based on the tertile results (T1 < 3.9 mmol/L, 3.9 ≤ T2 < 4.5 mmol/L, and T3 ≥ 4.5 mmol/L), and used the middle blood K group as a reference value for comparison.

### Variables

The baseline variables measured for the first time within 24 hours of admission to the ICU were gathered from the MIMIC-IV database, including: (a) the general information: gender, age, and body mass index (BMI); (b) vital signs: heart rate (HR), respiration rate (RR), systolic blood pressure (SBP), and diastolic blood pressure (DBP); (c) laboratory indicators: hemoglobin (Hgb), platelets count, white blood cell (WBC) count, calcium (Ca), sodium (Na), chlorine (Cl), K, blood glucose (BG), creatinine (Cr), and blood urea nitrogen (BUN); (d) comorbidity diseases: myocardial infarct, congestive heart failure, cerebrovascular disease, chronic pulmonary disease, respiratory failure, liver disease, kidney disease, malignant cancer, diabetes, and infection; (e) interventions: mechanical ventilation, diuretics use (including furosemide, hydrochlorothiazide, indapamide and metolazone), vasoactive drugs use (including epinephrine, norepinephrine, dopamine, and dobutamine), and renal replacement therapy (RRT) use; (f) severity of illness: SOFA score, comorbidity index, and simplified acute physiology score II (Saps II); (g) other variables: the stages of AKI. The data extraction was performed using PostgreSQL software (v13.9.3) and Navicat Premium software (v16.0.6) through the execution of Structured Query Language (SQL).

### Outcome

The study outcome was ICU 30-day mortality, defined as survival or death status 30 days from ICU admission.

### Statistical analysis

Continuous variables were presented as mean ± standard deviation (SD) for normally distributed data or median with interquartile range (IQR) for non-normally distributed data. They were compared using either the one-way analysis of variance (ANOVA) test or Kruskal-Wallis rank-sum test. Further analysis was conducted using Tukey’s Honest Significant Difference (HSD) post hoc tests or Dunn’s post hoc tests with Bonferroni correction to identify specific inter-group differences. Categorical variables were presented as count with percentage and compared using the chi-square test (χ^2^). Further analysis was conducted using pairwise Chi-square tests with Bonferroni correction. We performed univariate analysis and then multivariate accelerated failure time (AFT) models analysis to evaluate for independent associations between admission baseline blood K levels associated with ICU 30-day mortality. Hazard ratios (HRs) and 95% confidence intervals (CI) are reported. After considering the previously published relevant literatures and the clinical significance, we further explored the relationship between admission baseline blood K levels associated with ICU 30-day mortality by adjusting relevant variables included age, sex, BMI, Hgb, BG, Cr, myocardial infarct, congestive heart failure, respiratory failure, kidney disease, malignant cancer, SOFA score, comorbidity index. Survival curves were plotted based on Kaplan–Meier and log-rank analyses test. In the subgroup analyses, we stratified the study population by age (<65, ≥65 years) [34], sex (male, female), Cr (<1.5, ≥1.5 mg/dl) [35], kidney disease, AKI stage, RRT use and vasoactive drugs use. Heterogeneity between subgroups was assessed by AFT models, and interactions between subgroups were examined by likelihood ratio tests. In sensitivity analyses, we used clinical cut-off groupings for multivariate analyses and analyzed data before excluding missing values to further explore the relationship between admission baseline blood K and ICU 30-day mortality.

The statistical software packages R 3.3.2 (http://www.R-project.org, The R Foundation) and Free Statistics software versions 1.7.1 were used for all analyses. *P* <0.05 (two-tailed test) was considered statistically significant.

## Results

### Baseline characteristics

**Fig 1:**
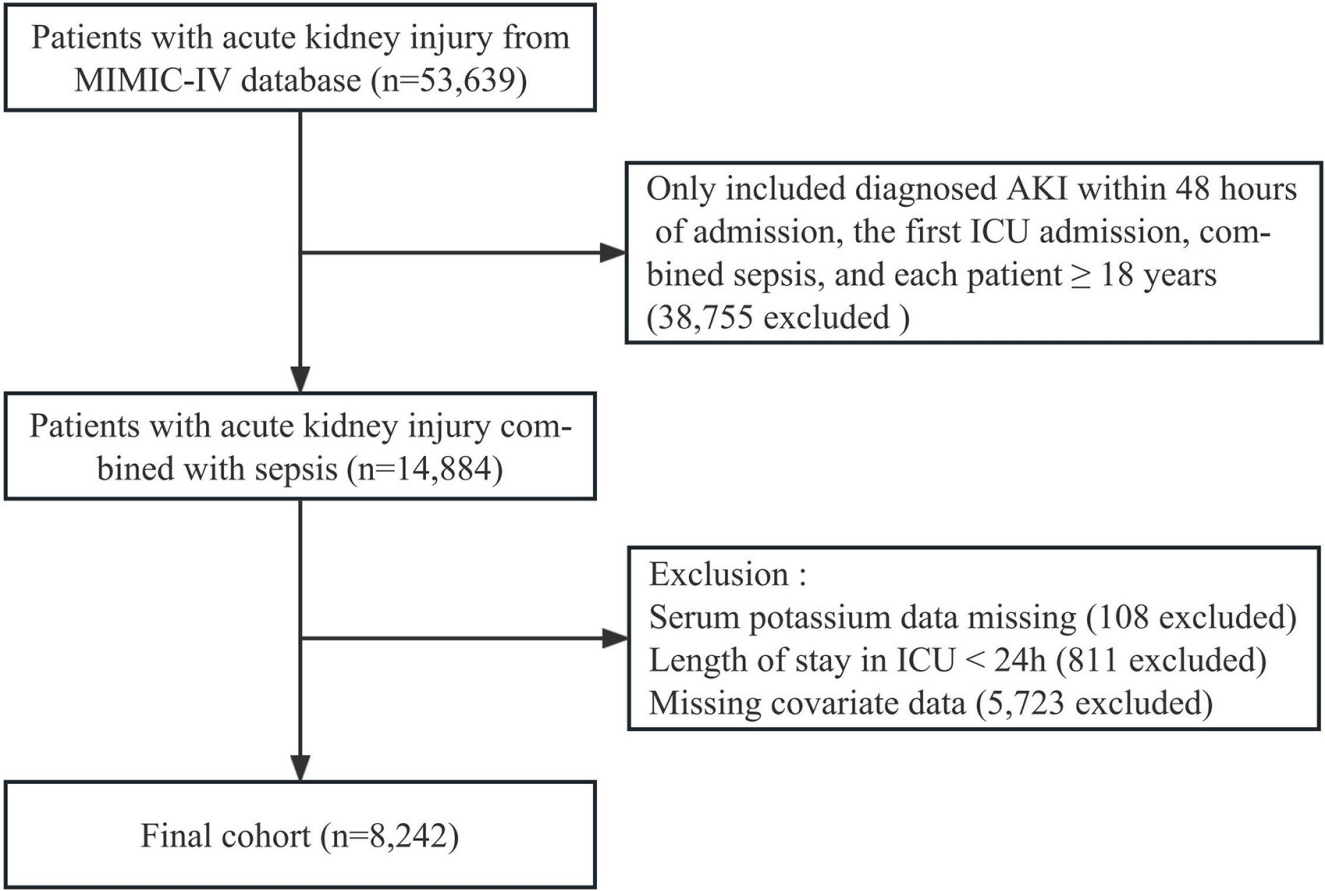
Flowchart of participant selection.

As shown in Fig 1, a total of 8,242 patients were ultimately included. Table 1 showed that 20.4% (n = 1,679) of these patients died during ICU 30-day stay. Of the 8,242 patients, 59.7% were male participants. Mean age was 65.2 ± 15.5 years. The prevalence of comorbidities among these patients was as follows: myocardial infarct (20.6%), congestive heart failure (33.6%), cerebrovascular disease (14.9%), chronic pulmonary disease (27.5%), respiratory failure (48.3%), liver disease (16.9%), kidney disease (23.7%), malignant cancer (12.2%), diabetes (32.6%) and infection (31.5%). Overall, the patients with higher blood K levels (≥ 4.5 mmol/L) were found to have higher values for BMI, WBC, BG, Cr, and BUN, as well as a higher representation of myocardial infarct, congestive heart failure, chronic pulmonary disease, respiratory failure, liver disease, kidney disease, malignant cancer, diabetes, SOFA score, Saps II, vasoactive drugs and RRT use than those in the other groups. Moreover, the patients with lower blood K levels (< 3.9 mmol/L) were found to have a higher incidence of cerebrovascular disease and infection. Detailed results of the comparisons between groups are presented in Table 1 and S1.

**Table 1:** Baseline characteristics of participants.

Variables	Total (n = 8,242) Mean±SD/Median (IQR)	K (mmol/L)			*p*-value
		T1 < 3.9 (n = 2,373)	3.9 ≤ T2 < 4.5 (n = 2,914)	T3 ≥ 4.5 (n = 2,955)	
Sex, Male, n (%)	4924 (59.7)	1200 (50.6)	1775 (60.9)	1949 (66.0)	< 0.001^abc^
Age (years)	65.2 ± 15.5	64.5 ± 16.5	65.6 ± 15.3	65.4 ± 14.9	0.032^a^
BMI (kg/m^2^)	30.3 ± 8.2	29.6 ± 8.1	30.3 ± 7.9	30.9 ± 8.6	< 0.001^abc^
HR (bmp)	107.1 ± 21.9	107.9 ± 21.3	106.4 ± 22.1	107.3 ± 22.1	0.038^a^
RR (bmp)	28.6 ± 6.6	28.6 ± 6.8	28.5 ± 6.6	28.8 ± 6.4	0.233
SBP (mmHg)	147.9 ± 24.1	148.7 ± 24.7	148.1 ± 24.0	147.1 ± 23.5	0.053
DBP (mmHg)	86.2 ± 20.3	86.9 ± 20.2	85.6 ± 20.3	86.1 ± 20.3	0.063
Laboratory tests					
Hgb (g/dL)	9.8 ± 2.2	9.9 ± 2.2	9.9 ± 2.1	9.6 ± 2.3	< 0.001^bc^
Platelets (×10^9/L)	155.0 (105.0, 217.0)	154.0 (102.0, 216.0)	157.0 (110.0, 217.0)	154.0 (103.0, 219.0)	0.032^a^
WBC (×10^9/L)	14.8 (10.7, 19.8)	14.0 (10.1, 19.3)	14.6 (10.7, 19.1)	15.5 (11.2, 20.8)	< 0.001^bc^
Ca (mmol/L)	7.9 ± 0.9	7.8 ± 0.9	8.0 ± 0.8	7.9 ± 0.9	< 0.001^abc^
Na (mmol/L)	136.6 ± 5.3	137.1 ± 5.6	137.0 ± 4.9	135.7 ± 5.4	< 0.001^bc^
Cl (mmol/L)	101.9 ± 6.6	101.9 ± 7.1	102.7 ± 6.1	101.1 ± 6.7	< 0.001^abc^
K (mmol/L)	4.3 ± 0.8	3.5 ± 0.3	4.1 ± 0.2	5.1 ± 0.7	< 0.001^abc^
BG (mg/dL)	155.7 ± 77.1	151.2 ± 78.4	151.5 ± 65.4	163.5 ± 85.7	< 0.001^bc^
Cr (mg/dL)	1.1 (0.8, 1.7)	0.9 (0.7, 1.4)	1.0 (0.8, 1.5)	1.4 (1.0, 2.4)	< 0.001^abc^
BUN (mg/dL)	22.0 (15.0, 36.0)	18.0 (13.0, 28.0)	20.0 (14.0, 31.0)	28.0 (18.0, 48.0)	< 0.001^abc^
Comorbidity diseases, n (%)					
Myocardial infarct	1697 (20.6)	414 (17.4)	612 (21.0)	671 (22.7)	< 0.001^ab^
Congestive heart failure	2771 (33.6)	721 (30.4)	949 (32.6)	1101 (37.3)	< 0.001^bc^
Cerebrovascular disease	1225 (14.9)	417 (17.6)	439 (15.1)	369 (12.5)	< 0.001^abc^
Chronic pulmonary disease	2263 (27.5)	603 (25.4)	758 (26.0)	902 (30.5)	< 0.001^bc^
Respiratory failure	3981 (48.3)	1170 (49.3)	1286 (44.1)	1525 (51.6)	< 0.001^ac^
Liver disease	1389 (16.9)	409 (17.2)	407 (14.0)	573 (19.4)	< 0.001^ac^
Kidney disease	1955 (23.7)	399 (16.8)	611 (21.0)	945 (32.0)	< 0.001^abc^
Malignant cancer	1005 (12.2)	260 (11.0)	347 (11.9)	398 (13.5)	0.017^b^
Diabetes	2687 (32.6)	620 (26.1)	952 (32.7)	1115 (37.7)	< 0.001^abc^
Infection	2596 (31.5)	804 (33.9)	869 (29.8)	923 (31.2)	0.006^a^
AKI stage, no (%)					< 0.001^bc^
1	2037 (24.7)	598 (25.2)	780 (26.8)	659 (22.3)	
2	3952 (47.9)	1228 (51.7)	1473 (50.5)	1251 (42.3)	
3	2253 (27.3)	547 (23.1)	661 (22.7)	1045 (35.4)	
Severity of illness					
SOFA score	3.0 (2.0, 5.0)	3.0 (2.0, 5.0)	3.0 (2.0, 5.0)	4.0 (2.0, 5.0)	< 0.001^bc^
Comorbidity index	6.0 (4.0, 8.0)	5.0 (4.0, 7.0)	6.0 (4.0, 8.0)	6.0 (4.0, 8.0)	< 0.001^abc^
Saps II	43.3 ± 14.8	41.5 ± 13.9	40.8 ± 14.0	47.3 ± 15.4	< 0.001^bc^
Interventions (day 1), n (%)					
Mechanical ventilation	7281 (88.3)	2058 (86.7)	2592 (88.9)	2631 (89.0)	0.015^ab^
Diuretics use	6291 (76.3)	1745 (73.5)	2284 (78.4)	2262 (76.5)	< 0.001^ab^
Vasoactive drugs use	5454 (66.2)	1496 (63.0)	1921 (65.9)	2037 (68.9)	< 0.001^bc^
RRT use	922 (11.2)	199 (8.4)	235 (8.1)	488 (16.5)	< 0.001^bc^
Outcomes, n (%)					
ICU 30-day mortality	1679 (20.4)	513 (21.6)	462 (15.9)	704 (23.8)	< 0.001^bc^

“a” represents that the comparison between Group T1 and Group T2 has a Bonferroni adjusted *p*-value < 0.05. “b” represents that the comparison between Group T1 and Group T3 has a Bonferroni adjusted *p*-value < 0.05. “c” represents that the comparison between Group T2 and Group T3 has a Bonferroni adjusted *p*-value < 0.05. BMI, body mass index; HR, heart rate; RR, respiration rate; SBP, systolic blood pressure; DBP, diastolic blood pressure; Hgb, hemoglobin; WBC, white blood cell; Ca, calcium; Na, sodium; Cl, chlorine; K, potassium; BG, blood glucose; Cr, creatinine; BUN, blood urea nitrogen; SOFA, sequential organ failure assessment; Saps II, simplified acute physiology score II; RRT, renal replacement therapy.

### Association between admission baseline blood K levels and ICU 30-day mortality

**Fig 2:**
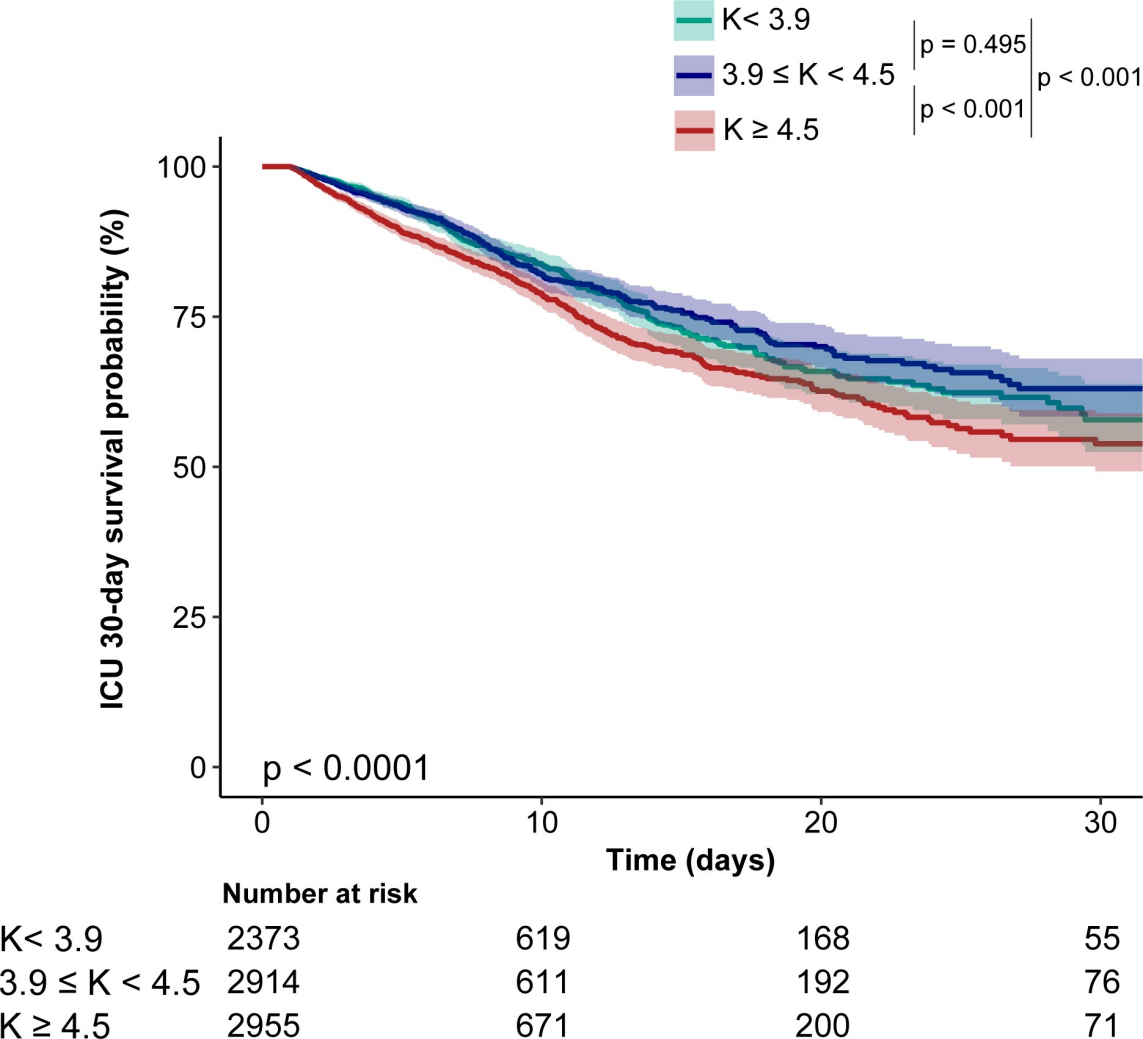
Kaplan–Meier survival curves for ICU 30-day mortality.

Kaplan-Meier curve in Fig 2 showed that patients with the higher blood K levels (≥ 4.5 mmol/L, T3) experienced higher mortality within 30 days of ICU admission, whereas those with middle blood K levels (3.9mmol/L ≤ K < 4.5 mmol/L, T2) had lower mortality rates (Log-rank test: *p* < 0.0001). The results of univariate analyses of various covariates and outcomes are shown in S2 Table. To assess the independent impact of admission baseline blood K levels on ICU 30-day mortality, three multivariate AFT models were constructed. HRs and 95% CIs were presented in Table 2. The results showed that each 1 mmol/L rise in blood K levels was associated with 13% (95% CI 1.06~1.20) increase in the risk of ICU 30-day mortality when as a continuous variable (model 3). In the extended multivariable AFT models, compared to the middle blood K group, the high blood K group (≥ 4.5 mmol/L) consistently showed significantly higher HRs across all three models (HRs range 1.22–1.34, *p* < 0.05), whereas the low blood K group (< 3.9 mmol/L) did not show significantly differences in HRs in all three models (HRs range 1.03–1.06, *p* > 0.05).

**Table 2:** Multivariable-adjust HRs and 95%CI of admission baseline blood K levels associated with ICU 30-day mortality.

Variables	Unadjusted		Model 1		Model 2		Model 3	
	HR (95%CI)	*p*-value	HR (95%CI)	*p*-value	HR (95%CI)	*p*-value	HR (95%CI)	*p*-value
K (continuous)	1.19 (1.13~1.26)	<0.001	1.19 (1.13~1.27)	<0.001	1.14 (1.07~1.21)	<0.001	1.13 (1.06~1.20)	<0.001
K (tertiles)								
T1 (< 3.9)	1.03 (0.90~1.17)	0.660	1.05 (0.93~1.19)	0.420	1.06 (0.94~1.21)	0.347	1.06 (0.93~1.21)	0.385
T2 (3.9~4.5)	ref		ref		ref		ref	
T3 (≥ 4.5)	1.34 (1.19~1.51)	<0.001	1.34 (1.19~1.51)	<0.001	1.24 (1.10~1.40)	<0.001	1.22 (1.08~1.38)	0.002
*P* for trend		<0.001		<0.001		0.001		0.005

Model 1 adjust for age and sex.

Model 2 adjust for Model 1 + BMI, Hgb, BG, Cr.

Model 3 adjust for Model 1 + Model 2 + myocardial infarct, congestive heart failure, respiratory failure, kidney disease, malignant cancer, SOFA score, comorbidity index.

BMI, body mass index; Hgb, hemoglobin; BG, blood glucose; Cr, creatinine; SOFA, sequential organ failure assessment.

### Sensitivity analysis

**Fig 3:**
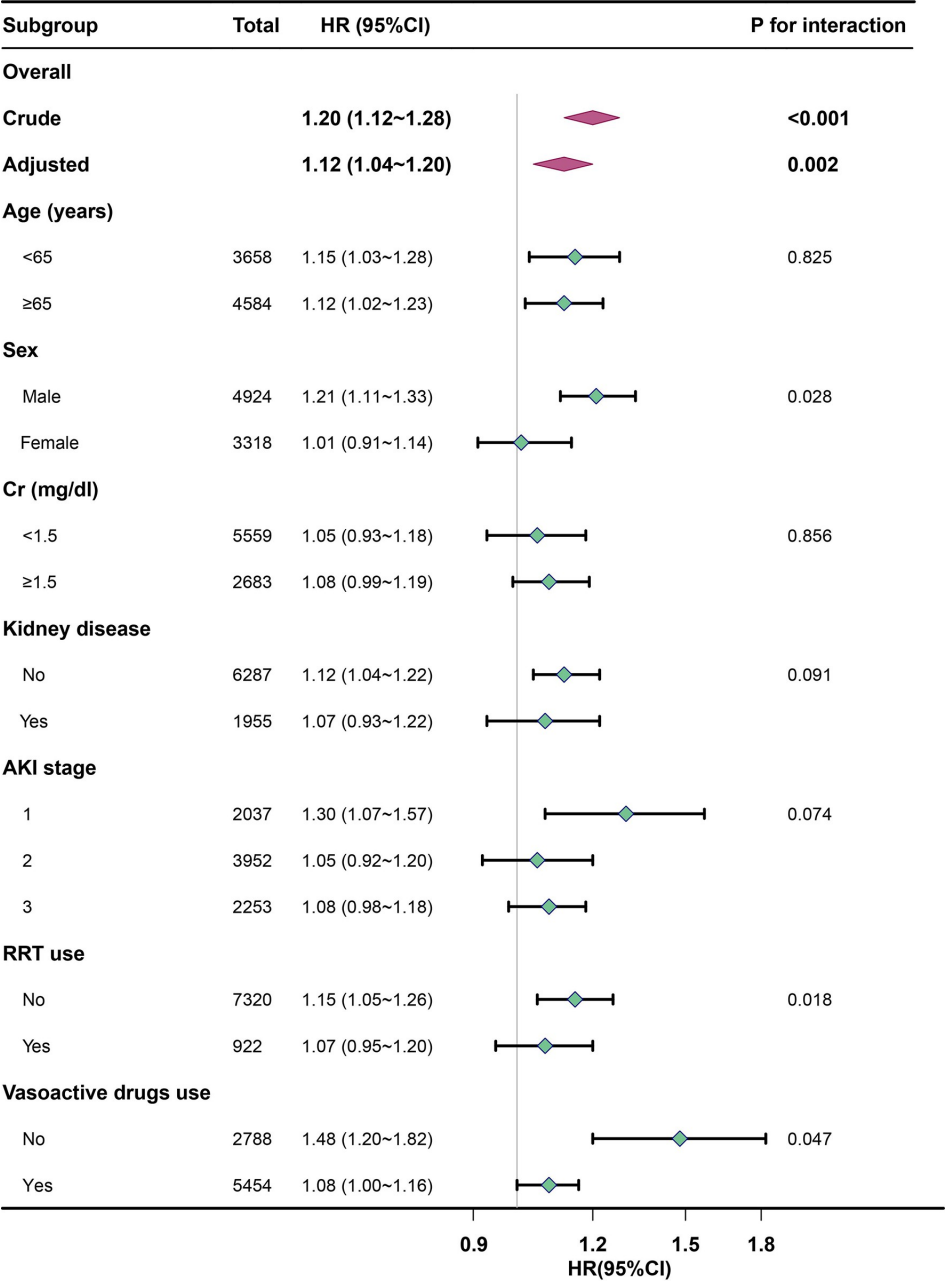
Subgroup analyses of admission baseline blood K levels associated with ICU 30-day mortality. Hazard ratios (HRs) were adjusted for age, sex, BMI, Hgb, BG, Cr, myocardial infarct, congestive heart failure, respiratory failure, kidney disease, malignant cancer, SOFA score, comorbidity index. BMI, body mass index; Hgb, hemoglobin; BG, blood glucose; Cr, creatinine; SOFA, sequential organ failure assessment.

As shown in Fig 3, according to the confounders including age, sex, Cr, kidney disease, AKI stage, RRT use, and vasoactive drugs use to perform subgroup analysis and to map forests. We observed possible interactions of sex, RRT and vasoactive drugs use subgroups (*p*-value for interaction < 0.05). Specifically, being male, not undergoing RRT, and not using vasoactive drugs may increase the effect of blood K on ICU 30-day mortality in patients with AKI combined with sepsis. These interactions were only significant for male when blood K levels were categorized (*p*-value for the interaction < 0.05 in S3 Table; *p*-value for the interaction > 0.05 in S4 and S5 Tables).

To support our findings, sensitivity analyses were performed. As shown in S6 Table and S1 Fig, after categorizing admission baseline blood K levels according to the clinical cut-off values of 3.5 and 5.5 mmol/L, the results were stable. Additionally, S7 Table and S2 Fig showed that even after accounting for the 5,723 patients with missing values, our conclusions remain stable and reliable.

## Discussion

This research uncovered that critically ill patients with AKI combined with sepsis who exhibit high blood K levels upon admission face a significantly increased risk of all-cause mortality in the ICU. Specifically, patients with high admission baseline blood K levels (≥4.5 mmol/L) had a 1.22-fold higher risk of ICU all-cause mortality than those with normal blood K levels (3.9mmol/L ≤ K < 4.5 mmol/L). Even though the results of the subgroup analysis indicated that being male, not receiving RRT, and no vasoactive drugs could intensify the impact of blood K levels on ICU 30-day mortality in these patients, the principal findings of this study remained robust even after adjusting for other potential covariates and through several sensitivity analyses.

Potassium is a crucial electrolyte and its homeostatic imbalance can lead to differential disease progression and even subsequent life-threatening complications [36,37]. Researches have shown that various factors, such as the use of medications (such as diuretics, antibiotics, etc.), decreased estimated glomerular filtration rate (eGFR) due to acute or chronic kidney injury, acid-base balance disorders, abnormal glucose metabolism, sepsis-induced multi-organ damage, surgical trauma, are closely associated with the imbalance of potassium homeostasis in patients in the ICU [12,38–40]. For instance, Gao et al. [24] reported that hypokalemia (<3.7 mmol/L) or hyperkalemia (≥4.8 mmol/L) among critically ill patients with acute kidney injury was associated with the 90-day mortality. Very recently, Li et al. [23] demonstrated that blood K levels of 4.10–5.49 mmol/L and ≥ 5.50 mmol/L were associated with a significantly increased 90-day and 1-year risk of death in very elderly patients with AKI. Another study found that the patients with AKI receiving RRT in the ICU with blood K levels between 3.0 and 4.0 mmol/L had the lowest mortality rates [41]. Unfortunately, their research was limited to elderly patients or those receiving RRT, and the study outcomes were related to 90-day or 1-year mortality rates. However, they did not extensively explore the short-term survival outcomes among AKI patients with sepsis comorbidity. Unexpectedly, in our study population, ICU 30-day mortality significantly increased in patients with higher blood K levels (≥4.5 mmol/L) compared to medium blood K levels (3.9–4.5 mmol/L) and lower blood K levels (< 3.9 mmol/L). This suggests that early attention to blood K levels may help reduce mortality rates in AKI patients with sepsis comorbidity.

The standard clinical range for blood K levels (3.5–5.5 mmol/L) is primarily derived from data on healthy individuals, and it remains unknown whether these values apply to AKI patients with sepsis comorbidity [42]. However, our results have shown that blood K levels above 4.5 mmol/L were associated with higher mortality, which means even patients with normal blood K levels (4.5–5.5 mmol/L) can have increased mortality risk. This observation highlights a potential oversight in clinical practice where strict electrolyte management may be neglected for patients whose blood potassium levels fall within this "normal" range, potentially contributing to higher mortality rates among critically ill individuals [23,43]. It is worth noting that for critically ill ICU patients with hyperkalemia, careful assessment of volume status and appropriate fluid resuscitation are also crucial parts of treatment [44]. Considering the risk of Major Adverse Kidney Events by day 30 (MAKE30), including persistent renal dysfunction and the need for renal replacement therapy, early optimization of fluid balance helps to prevent further kidney injury and improve patient prognosis [45]. This finding also serves as a reminder for clinicians to assess other conditions in the body of AKI patients with sepsis comorbidity, even if their blood K levels are within the normal range. It is important to thoroughly evaluate and optimize the treatment accordingly. Interestingly, as shown in Fig 2, our study found that the ICU 30-day survival rate was slightly lower in the lower blood K levels group than in the medium blood K levels group, yet no significant difference in mortality was detected between these groups after adjusting for confounders (95%CI 0.93 ~ 1.21, *p*-value > 0.05). We proposed two possible explanations for this finding: i) Studies have demonstrated significant impacts of hypokalemia on increased overall mortality in dialysis, and congestive heart failure patients [46,47]. The limited presence of such diagnoses in our study’s low potassium group could have diminished the observed effect of low potassium levels on mortality. ii) Hypokalemia can lead to arrhythmias and impaired cardiac function, which may increase the long-term risk of adverse cardiac events and cardiovascular-related death in critically ill patients [48,49]. Nonetheless, this relationship warrants further investigation through extended and more in-depth research to establish a definitive relationship.

The treatment and prevention of AKI combined with sepsis remains a major challenge to the intensive care physicians [50,51]. The onset of this condition is believed to be associated with multi-mechanisms, such as endothelial dysfunction, renal microcirculation disorder, renal cell cycle stress, tubular injury, mitochondrial injury and inflammation [52–56]. Hyperkalemia was a common electrolyte abnormality among critically ill patients [57]. Elevated blood K levels not only lead to abnormal electrical activity of the heart, increasing the risk of arrhythmias [58], but they can also promote muscle paralysis, including the respiratory muscles, which in turn have an impact on the patient’s ventilatory function [59]. In addition, hyperkalemia exacerbates sepsis-mediated inflammatory cytokine release and hemodynamic alterations, further deteriorating renal function in patients with AKI [59,60]. As found in our study, higher admission baseline blood K levels was independently associated with ICU 30-day mortality in patients with AKI combined with sepsis. Although sex, the use of RRT and vasoactive drugs showed possible interactions in subgroup analyses, these were significantly pronounced for sex when blood K levels were categorized. These findings may indicate the necessity for more rigorous management of blood K levels in male patients in the ICU.

This is the first study, to our knowledge, examining the association between admission baseline blood K levels and ICU 30-day mortality in among patients with AKI combined with sepsis. In this study, blood K levels were analyzed both as a continuous variable and a categorical variable. Various methods including multifactorial regression, model adjustment, and subgroup analysis were employed to confirm the association between higher admission baseline blood K levels and ICU 30-day mortality. Likewise, our principal findings remained consistent in analyses employing clinical cut-off values and prior to the exclusion of missing data, emphasizing the robustness and reliability of our results.

There were also several limitations in our study. Firstly, this is an observational study, we were only able to establish an association between variables rather than a causal relationship. Further randomized controlled trial research is needed to better understand the relationship between blood K levels, comorbidities, and mortality outcomes. Secondly, due to the inherent nature of retrospective cohort studies, multiple confounding factors are inevitable. Thus, we conducted multifactorial analyses to adjust for the effect of confounding factors on the study outcome as much as possible. Thirdly, we only analyzed baseline blood K levels on patients first admitted to the ICU in the database, future studies could also explore the impact of serum sodium levels and other electrolytes on patient outcomes. Additionally, longitudinal studies tracking changes in blood K levels and other biomarkers throughout hospitalization would offer insights into the dynamic nature of these associations. Fourthly, we analyzed the influence of the use of vasoactive drugs and RRT, but the use of renin-angiotensin system (RAS) inhibitors was not included. In fact, the treatment of RAS inhibitors often disturbs blood K homeostasis. Fifthly, we lack specific data pertaining to adverse events directly associated with high or low blood K levels, including treatment-related arrhythmias. Finally, it is necessary to conduct comprehensive in vitro and in vivo investigations to elucidate the biological mechanisms that underpin the relationship between potassium dysregulation and mortality in the context of AKI and sepsis. Further insights into these mechanisms will be invaluable for understanding the pathophysiology of AKI and sepsis and may reveal potential therapeutic targets.

## Conclusion

In this study for patients with AKI combined with sepsis in ICU, our findings revealed a relationship between the admission baseline blood K levels and all-cause mortality. Briefly, the mortality increased significantly as blood K exceeded 4.5 mmol/L. More careful and timely correction of blood K disturbances may be an effective measure to improve the prognosis of these patients.

## Supporting information

S1 Table Post hoc tests for inter-group comparisons. BMI, body mass index; HR, heart rate; Hgb, hemoglobin; WBC, white blood cell; Ca, calcium; Na, sodium; Cl, chlorine; K, potassium; BG, blood glucose; Cr, creatinine; BUN, blood urea nitrogen; SOFA, sequential organ failure assessment; Saps II, simplified acute physiology score II; RRT, renal replacement therapy.
(DOCX)

S2 Table Results of univariate analysis of ICU 30-day mortality. ✝: The continuous variables goes up per 10 units. ↗: The categorical variables are referenced by ‘No’. BMI, body mass index; HR, heart rate; RR, respiration rate; SBP, systolic blood pressure; DBP, diastolic blood pressure; Hgb, hemoglobin; WBC, white blood cell; Ca, calcium; Na, sodium; Cl, chlorine; K, potassium; BG, blood glucose; Cr, creatinine; BUN, blood urea nitrogen; SOFA, sequential organ failure assessment; Saps II, simplified acute physiology score II; RRT, renal replacement therapy.
(DOCX)

S3 Table Interactive effect of blood K levels and ICU 30-day mortality in patients male and female. Hazard ratios (HRs) were adjusted for age, sex, BMI, Hgb, BG, Cr, myocardial infarct, congestive heart failure, respiratory failure, kidney disease, malignant cancer, SOFA score, comorbidity index. BMI, body mass index; Hgb, hemoglobin; BG, blood glucose; Cr, creatinine; SOFA, sequential organ failure assessment.
(DOCX)

S4 Table Interactive effect of blood K levels and ICU 30-day mortality in patients with and without RRT use. Hazard ratios (HRs) were adjusted for age, sex, BMI, Hgb, BG, Cr, myocardial infarct, congestive heart failure, respiratory failure, kidney disease, malignant cancer, SOFA score, comorbidity index. BMI, body mass index; Hgb, hemoglobin; BG, blood glucose; Cr, creatinine; SOFA, sequential organ failure assessment.
(DOCX)

S5 Table Interactive effect of blood K levels and ICU 30-day mortality in patients with and without vasoactive drugs use. Hazard ratios (HRs) were adjusted for age, sex, BMI, Hgb, BG, Cr, myocardial infarct, congestive heart failure, respiratory failure, kidney disease, malignant cancer, SOFA score, comorbidity index. BMI, body mass index; Hgb, hemoglobin; BG, blood glucose; Cr, creatinine; SOFA, sequential organ failure assessment.
(DOCX)

S6 Table Multivariable-adjust HRs and 95%CI of blood K levels associated with ICU 30-day mortality (grouped according to clinical cut-off values). Model 1 adjust for age and sex. Model 2 adjust for Model 1 + BMI, Hgb, BG, Cr. Model 3 adjust for Model 1 + Model 2 + myocardial infarct, congestive heart failure, respiratory failure, kidney disease, malignant cancer, SOFA score, comorbidity index. BMI, body mass index; Hgb, hemoglobin; BG, blood glucose; Cr, creatinine; SOFA, sequential organ failure assessment.
(DOCX)

S7 Table Multivariable-adjust HRs and 95%CI of blood K levels associated with ICU 30-day mortality (before excluding missing values). Model 1 adjust for age and sex. Model 2 adjust for Model 1 + BMI, Hgb, BG, Cr. Model 3 adjust for Model 1 + Model 2 + myocardial infarct, congestive heart failure, respiratory failure, kidney disease, malignant cancer, SOFA score, comorbidity index. BMI, body mass index; Hgb, hemoglobin; BG, blood glucose; Cr, creatinine; SOFA, sequential organ failure assessment.
(DOCX)

S1 Fig Kaplan–Meier survival curves for ICU 30-day mortality (grouped according to clinical cut-off values). (DOCX)

S2 Fig Kaplan–Meier survival curves for ICU 30-day mortality (before excluding missing values). (DOCX)

## Abbreviations

AFT: Accelerated Failure Time
AKI: Acute Kidney Injury
ANOVA: Analysis of Variance
BG: Blood Glucose
BMI: Body Mass Index
BUN: Blood Urea Nitrogen
Ca: Calcium
CITI: Collaborative Institutional Training Initiative
CI: Confidence Interval
Cl: Chlorine
Cr: Creatinine
DBP: Diastolic Blood Pressure
eGFR: Estimated Glomerular Filtration Rate
Hgb: Hemoglobin
HR: Hazard Ratio
HR: Heart Rate
HSD: Honest Significant Difference
ICU: Intensive Care Unit
IQR: Interquartile Range
KDIGO: Kidney Disease: Improving Global Outcomes
K: Potassium
MAKE30: Major Adverse Kidney Events by day 30
MIMIC-IV: Medical Information Mart for Intensive Care IV
Na: Sodium
RAS: Renin-angiotensin System
RRT: Renal Replacement Therapy
RR: Respiration Rate
Saps II: Simplified Acute Physiology Score II
SBP: Systolic Blood Pressure
SD: Standard Deviation
SOFA: Sequential Organ Failure Assessment
SQL: Structured Query Language
STROBE: Strengthening the Reporting of Observational Studies in Epidemiology
T: Tertile
WBC: White Blood Cell

## References

[1] Zuk A, Bonventre JV. Acute Kidney Injury. Annual review of medicine. 2016; 67:293–307. doi: 10.1146/annurev-med-050214-013407 . 26768243 PMC4845743

[2] Hoste EA, Bagshaw SM, Bellomo R, Cely CM, Colman R, Cruz DN, et al. Epidemiology of acute kidney injury in critically ill patients: the multinational AKI-EPI study. Intensive care medicine. 2015;41(8):1411–23. doi: 10.1007/s00134-015-3934-7 . 26162677

[3] Griffin BR, Liu KD, Teixeira JP. Critical Care Nephrology: Core Curriculum 2020. American journal of kidney diseases: the official journal of the National Kidney Foundation. 2020;75(3):435–52. doi: 10.1053/j.ajkd.2019.10.010 . 31982214 PMC7333544

[4] Peerapornratana S, Manrique-Caballero CL, Gómez H, Kellum JA. Acute kidney injury from sepsis: current concepts, epidemiology, pathophysiology, prevention and treatment. Kidney international. 2019;96(5):1083–99. doi: 10.1016/j.kint.2019.05.026 . 31443997 PMC6920048

[5] Silver SA, Chertow GM. The Economic Consequences of Acute Kidney Injury. Nephron. 2017;137(4):297–301. doi: 10.1159/000475607 . 28595193 PMC5743773

[6] Rhee C, Dantes R, Epstein L, Murphy DJ, Seymour CW, Iwashyna TJ, et al. Incidence and Trends of Sepsis in US Hospitals Using Clinical vs Claims Data, 2009–2014. Jama. 2017;318(13):1241–9. doi: 10.1001/jama.2017.13836 . 28903154 PMC5710396

[7] Coca SG, Yusuf B, Shlipak MG, Garg AX, Parikh CR. Long-term risk of mortality and other adverse outcomes after acute kidney injury: a systematic review and meta-analysis. American journal of kidney diseases: the official journal of the National Kidney Foundation. 2009;53(6):961–73. doi: 10.1053/j.ajkd.2008.11.034 . 19346042 PMC2726041

[8] Sun S, Chen R, Dou X, Dai M, Long J, Wu Y, et al. Immunoregulatory mechanism of acute kidney injury in sepsis: A Narrative Review. Biomedicine & pharmacotherapy = Biomedecine & pharmacotherapie. 2023; 159:114202. doi: 10.1016/j.biopha.2022.114202 . 36621143

[9] White KC, Serpa-Neto A, Hurford R, Clement P, Laupland KB, See E, et al. Sepsis-associated acute kidney injury in the intensive care unit: incidence, patient characteristics, timing, trajectory, treatment, and associated outcomes. A multicenter, observational study. Intensive care medicine. 2023. doi: 10.1007/s00134-023-07138-0 . 37432520 PMC10499944

[10] Kellum JA, Romagnani P, Ashuntantang G, Ronco C, Zarbock A, Anders HJ. Acute kidney injury. Nature reviews Disease primers. 2021;7(1):52. doi: 10.1038/s41572-021-00284-z . 34267223

[11] Palmer BF, Clegg DJ. Physiology and Pathophysiology of Potassium Homeostasis: Core Curriculum 2019. American journal of kidney diseases: the official journal of the National Kidney Foundation. 2019;74(5):682–95. doi: 10.1053/j.ajkd.2019.03.427 . 31227226

[12] Kettritz R, Loffing J. Potassium homeostasis—Physiology and pharmacology in a clinical context. Pharmacology & therapeutics. 2023; 249:108489. doi: 10.1016/j.pharmthera.2023.108489 . 37454737

[13] Sumida K, Dashputre AA, Potukuchi PK, Thomas F, Obi Y, Molnar MZ, et al. Laxative Use and Risk of Dyskalemia in Patients with Advanced CKD Transitioning to Dialysis. Journal of the American Society of Nephrology: JASN. 2021;32(4):950–9. doi: 10.1681/ASN.2020081120 . 33547216 PMC8017552

[14] McDonough AA, Youn JH. Potassium Homeostasis: The Knowns, the Unknowns, and the Health Benefits. Physiology (Bethesda, Md). 2017;32(2):100–11. doi: 10.1152/physiol.00022.2016 . 28202621 PMC5337831

[15] Yamada S, Inaba M. Potassium Metabolism and Management in Patients with CKD. Nutrients. 2021;13(6). doi: 10.3390/nu13061751 . 34063969 PMC8224083

[16] Winsløw U, Sakthivel T, Zheng C, Bosselmann H, Haugan K, Bruun N, et al. Targeted potassium levels to decrease arrhythmia burden in high risk patients with cardiovascular diseases (POTCAST): Study protocol for a randomized controlled trial. American heart journal. 2022; 253:59–66. doi: 10.1016/j.ahj.2022.07.003 . 35835265

[17] Dixon DL, Abbate A. Potassium levels in acute myocardial infarction: definitely worth paying attention to. European heart journal Cardiovascular pharmacotherapy. 2015;1(4):252–3. doi: 10.1093/ehjcvp/pvv029 . 27532448

[18] McLean A, Nath M, Sawhney S. Population Epidemiology of Hyperkalemia: Cardiac and Kidney Long-term Health Outcomes. American journal of kidney diseases: the official journal of the National Kidney Foundation. 2022;79(4):527–38.e1. doi: 10.1053/j.ajkd.2021.07.008 . 34419518

[19] Genovesi S, Regolisti G, Burlacu A, Covic A, Combe C, Mitra S, et al. The conundrum of the complex relationship between acute kidney injury and cardiac arrhythmias. Nephrology, dialysis, transplantation: official publication of the European Dialysis and Transplant Association—European Renal Association. 2023;38(5):1097–112. doi: 10.1093/ndt/gfac210 . 35777072

[20] Regolisti G, Maggiore U, Greco P, Maccari C, Parenti E, Di Mario F, et al. Electrocardiographic T wave alterations and prediction of hyperkalemia in patients with acute kidney injury. Internal and emergency medicine. 2020;15(3):463–72. doi: 10.1007/s11739-019-02217-x . 31686358

[21] An JN, Lee JP, Jeon HJ, Kim DH, Oh YK, Kim YS, et al. Severe hyperkalemia requiring hospitalization: predictors of mortality. Critical care (London, England). 2012;16(6): R225. doi: 10.1186/cc11872 . 23171442 PMC3672605

[22] Chávez-Íñiguez JS, Maggiani-Aguilera P, Aranda-García de Quevedo A, Claure-Del Granado R, Vega-Vega O, López-Giacoman SR, et al. Serum Potassium Trajectory during Acute Kidney Injury and Mortality Risk. Nephron. 2023;147(9):521–30. doi: 10.1159/000529588 . 36808092

[23] Li Q, Li Y, Zhou F. Association of serum potassium level with early and late mortality in very elderly patients with acute kidney injury. Journal of intensive medicine. 2022;2(1):50–5. doi: 10.1016/j.jointm.2021.11.005 . 36789231 PMC9923985

[24] Gao XP, Zheng CF, Liao MQ, He H, Liu YH, Jing CX, et al. Admission serum sodium and potassium levels predict survival among critically ill patients with acute kidney injury: a cohort study. BMC nephrology. 2019;20(1):311. doi: 10.1186/s12882-019-1505-9 . 31395027 PMC6686448

[25] Zarbock A, Nadim MK, Pickkers P, Gomez H, Bell S, Joannidis M, et al. Sepsis-associated acute kidney injury: consensus report of the 28th Acute Disease Quality Initiative workgroup. Nature reviews Nephrology. 2023;19(6):401–17. doi: 10.1038/s41581-023-00683-3 . 36823168

[26] Ahmad MS, Ahmad D, Medhat N, Zaidi SAH, Farooq H, Tabraiz SA. Electrolyte Abnormalities in Neonates with Probable and Culture-Proven Sepsis and its Association with Neonatal Mortality. Journal of the College of Physicians and Surgeons—Pakistan: JCPSP. 2018;28(3):206–9. doi: 10.29271/jcpsp.2018.03.206 . 29544577

[27] Su K, Li XT, Hong FX, Jin M, Xue FS. Lidocaine pretreatment attenuates inflammatory response and protects against sepsis-induced acute lung injury via inhibiting potassium efflux-dependent NLRP3 activation. Inflammation research: official journal of the European Histamine Research Society [et al]. 2023;72(12):2221–35. doi: 10.1007/s00011-023-01810-3 . 37930383

[28] Ogawa Y, Ezaki S, Shimojo N, Kawano S. Case Report: Reduced CSF Orexin Levels in a Patient with Sepsis. Frontiers in neuroscience. 2021; 15:739323. doi: 10.3389/fnins.2021.739323 . 34690677 PMC8526783

[29] Drumheller BC, Tuffy E, Gibney F, Stallard S, Siewers C, Korvek S. Severe bradycardia from severe hyperkalemia: Patient characteristics, outcomes and factors associated with hemodynamic support. The American journal of emergency medicine. 2022;55: 117–25. doi: 10.1016/j.ajem.2022.03.007 . 35306438

[30] Zhou S, Zeng Z, Wei H, Sha T, An S. Early combination of albumin with crystalloids administration might be beneficial for the survival of septic patients: a retrospective analysis from MIMIC-IV database. Annals of intensive care. 2021;11(1):42. doi: 10.1186/s13613-021-00830-8 . 33689042 PMC7947075

[31] von Elm E, Altman DG, Egger M, Pocock SJ, Gøtzsche PC, Vandenbroucke JP. The Strengthening the Reporting of Observational Studies in Epidemiology (STROBE) Statement: guidelines for reporting observational studies. International journal of surgery (London, England). 2014;12(12):1495–9. doi: 10.1016/j.ijsu.2014.07.013 . 25046131

[32] Palevsky PM, Liu KD, Brophy PD, Chawla LS, Parikh CR, Thakar CV, et al. KDOQI US commentary on the 2012 KDIGO clinical practice guideline for acute kidney injury. American journal of kidney diseases: the official journal of the National Kidney Foundation. 2013;61(5):649–72. doi: 10.1053/j.ajkd.2013.02.349 . 23499048

[33] Singer M, Deutschman CS, Seymour CW, Shankar-Hari M, Annane D, Bauer M, et al. The Third International Consensus Definitions for Sepsis and Septic Shock (Sepsis-3). Jama. 2016;315(8):801–10. doi: 10.1001/jama.2016.0287 . 26903338 PMC4968574

[34] Schmitt R, Coca S, Kanbay M, Tinetti ME, Cantley LG, Parikh CR. Recovery of kidney function after acute kidney injury in the elderly: a systematic review and meta-analysis. American journal of kidney diseases: the official journal of the National Kidney Foundation. 2008;52(2):262–71. doi: 10.1053/j.ajkd.2008.03.005 . 18511164

[35] Floege J, Barbour SJ, Cattran DC, Hogan JJ, Nachman PH, Tang SCW, et al. Management and treatment of glomerular diseases (part 1): conclusions from a Kidney Disease: Improving Global Outcomes (KDIGO) Controversies Conference. Kidney international. 2019;95(2):268–80. doi: 10.1016/j.kint.2018.10.018 . 30665568

[36] Udensi UK, Tchounwou PB. Potassium Homeostasis, Oxidative Stress, and Human Disease. International journal of clinical and experimental physiology. 2017;4(3):111–22. doi: 10.4103/ijcep.ijcep_43_17 . 29218312 PMC5716641

[37] Kovesdy CP, Appel LJ, Grams ME, Gutekunst L, McCullough PA, Palmer BF, et al. Potassium Homeostasis in Health and Disease: A Scientific Workshop Cosponsored by the National Kidney Foundation and the American Society of Hypertension. American journal of kidney diseases: the official journal of the National Kidney Foundation. 2017;70(6):844–58. doi: 10.1053/j.ajkd.2017.09.003 . 29029808

[38] Dammann K, Timmons M, Edelman M, Pierce CA, Higdon E, Bernard AC. Electrolyte Analysis and Replacement: Challenging a Paradigm in Surgical Patients. Journal of trauma nursing: the official journal of the Society of Trauma Nurses. 2020;27(3):141–5. doi: 10.1097/JTN.0000000000000502 . 32371730

[39] Jiang Z, Bo L, Xu Z, Song Y, Wang J, Wen P, et al. An explainable machine learning algorithm for risk factor analysis of in-hospital mortality in sepsis survivors with ICU readmission. Computer methods and programs in biomedicine. 2021; 204:106040. doi: 10.1016/j.cmpb.2021.106040 . 33780889

[40] Clase CM, Carrero JJ, Ellison DH, Grams ME, Hemmelgarn BR, Jardine MJ, et al. Potassium homeostasis and management of dyskalemia in kidney diseases: conclusions from a Kidney Disease: Improving Global Outcomes (KDIGO) Controversies Conference. Kidney international. 2020;97(1):42–61. doi: 10.1016/j.kint.2019.09.018 . 31706619

[41] Mao IC, Lin PR, Wu SH, Hsu HH, Hung PS, Kor CT. First 24-Hour Potassium Concentration and Variability and Association with Mortality in Patients Requiring Continuous Renal Replacement Therapy in Intensive Care Units: A Hospital-Based Retrospective Cohort Study. Journal of clinical medicine. 2022;11(12). doi: 10.3390/jcm11123383 . 35743452 PMC9224685

[42] Polcwiartek C, Hansen SM, Kragholm K, Krogager ML, Aldahl M, Køber L, et al. Prognostic role of serum sodium levels across different serum potassium levels in heart failure patients: A Danish register-based cohort study. International journal of cardiology. 2018; 272:244–9. doi: 10.1016/j.ijcard.2018.08.045 . 30139700

[43] Zhang X, Wang M, Zhu Z, Qu H, Gu J, Ni T, et al. Serum potassium level, variability and in-hospital mortality in acute myocardial infarction. European journal of clinical investigation. 2022;52(7): e13772. doi: 10.1111/eci.13772 . 35294777

[44] Lee JW. Fluid and electrolyte disturbances in critically ill patients. Electrolyte & blood pressure: E & BP. 2010;8(2):72–81. doi: 10.5049/EBP.2010.8.2.72 . 21468200 PMC3043756

[45] Zampieri FG, Bagshaw SM, Semler MW. Fluid Therapy for Critically Ill Adults with Sepsis: A Review. Jama. 2023;329(22):1967–80. doi: 10.1001/jama.2023.7560 . 37314271

[46] Palaka E, Grandy S, Darlington O, McEwan P, van Doornewaard A. Associations between serum potassium and adverse clinical outcomes: A systematic literature review. International journal of clinical practice. 2020;74(1): e13421. doi: 10.1111/ijcp.13421 . 31532067

[47] Hoppe LK, Muhlack DC, Koenig W, Carr PR, Brenner H, Schöttker B. Association of Abnormal Serum Potassium Levels with Arrhythmias and Cardiovascular Mortality: A Systematic Review and Meta-Analysis of Observational Studies. Cardiovascular drugs and therapy. 2018;32(2):197–212. doi: 10.1007/s10557-018-6783-0 . 29679302

[48] Thongprayoon C, Cheungpasitporn W, Thirunavukkarasu S, Petnak T, Chewcharat A, Bathini T, et al. Serum Potassium Levels at Hospital Discharge and One-Year Mortality among Hospitalized Patients. Medicina (Kaunas, Lithuania). 2020;56(5). doi: 10.3390/medicina56050236 . 32423140 PMC7279137

[49] Sfairopoulos D, Arseniou A, Korantzopoulos P. Serum potassium and heart failure: association, causation, and clinical implications. Heart failure reviews. 2021;26(3):479–86. doi: 10.1007/s10741-020-10039-9 . 33098029

[50] White KC, Serpa-Neto A, Hurford R, Clement P, Laupland KB, See E, et al. Sepsis-associated acute kidney injury in the intensive care unit: incidence, patient characteristics, timing, trajectory, treatment, and associated outcomes. A multicenter, observational study. Intensive care medicine. 2023;49(9):1079–89. doi: 10.1007/s00134-023-07138-0 . 37432520 PMC10499944

[51] Godin M, Murray P, Mehta RL. Clinical approach to the patient with AKI and sepsis. Seminars in nephrology. 2015;35(1):12–22. doi: 10.1016/j.semnephrol.2015.01.003 . 25795496 PMC5617729

[52] Wang Y, Xi W, Zhang X, Bi X, Liu B, Zheng X, et al. CTSB promotes sepsis-induced acute kidney injury through activating mitochondrial apoptosis pathway. Frontiers in immunology. 2022; 13:1053754. doi: 10.3389/fimmu.2022.1053754 . 36713420 PMC9880165

[53] Tao X, Chen C, Luo W, Zhou J, Tian J, Yang X, et al. Combining renal cell arrest and damage biomarkers to predict progressive AKI in patient with sepsis. BMC nephrology. 2021;22(1):415. doi: 10.1186/s12882-021-02611-8 . 34906098 PMC8672478

[54] Poston JT, Koyner JL. Sepsis associated acute kidney injury. BMJ (Clinical research ed). 2019;364: k4891. doi: 10.1136/bmj.k4891 . 30626586 PMC6890472

[55] Lelubre C, Vincent JL. Mechanisms and treatment of organ failure in sepsis. Nature reviews Nephrology. 2018;14(7):417–27. doi: 10.1038/s41581-018-0005-7 . 29691495

[56] Ahmadian E, Hosseiniyan Khatibi SM, Razi Soofiyani S, Abediazar S, Shoja MM, Ardalan M, et al. Covid-19 and kidney injury: Pathophysiology and molecular mechanisms. Reviews in medical virology. 2021;31(3): e2176. doi: 10.1002/rmv.2176 . 33022818 PMC7646060

[57] Brueske B, Sidhu MS, Schulman-Marcus J, Kashani KB, Barsness GW, Jentzer JC. Hyperkalemia Is Associated with Increased Mortality Among Unselected Cardiac Intensive Care Unit Patients. Journal of the American Heart Association. 2019;8(7): e011814. doi: 10.1161/JAHA.118.011814 . 30922150 PMC6509722

[58] Rossignol P, Legrand M, Kosiborod M, Hollenberg SM, Peacock WF, Emmett M, et al. Emergency management of severe hyperkalemia: Guideline for best practice and opportunities for the future. Pharmacological research. 2016;113(Pt A):585–91. doi: 10.1016/j.phrs.2016.09.039 . 27693804

[59] Freeman SJ, Fale AD. Muscular paralysis and ventilatory failure caused by hyperkalaemia. British journal of anaesthesia. 1993;70(2):226–7. doi: 10.1093/bja/70.2.226 . 8435272

[60] Kindgen-Milles D. [Acute kidney injury in the perioperative setting]. Medizinische Klinik, Intensivmedizin und Notfallmedizin. 2014;109(5):324–30. doi: 10.1007/s00063-014-0348-1 . 24844158

