# Supplementary material for: Association between admission baseline blood potassium levels and all-cause mortality in patients with acute kidney injury combined with sepsis: A retrospective cohort study
Source: PLoS One. 2024 Nov 20;19(11):e0309764. doi: 10.1371/journal.pone.0309764 (PMC11578480; doi:10.1371/journal.pone.0309764)
Supplement: S1 Table — BMI, body mass index; HR, heart rate; Hgb, hemoglobin; WBC, white blood cell; Ca, calcium; Na, sodium; Cl, chlorine; K, potassium; BG, blood glucose; Cr, creatinine; BUN, blood urea nitrogen; SOFA, sequential organ failure assessment; Saps II, simplified acute physiology score II; RRT, renal replacement therapy. (DOCX) [file pone.0309764.s001.docx]

**S1 Table. Post hoc tests for inter-group comparisons.**

| **Variables** | **Post hoc tests adjusted *p*-value** | | |
| --- | --- | --- | --- |
|  | **T1 VS. T2** | **T1 VS. T3** | **T2 VS. T3** |
| Sex | <0.001 | <0.001 | <0.001 |
| Age | 0.032 | 0.108 | 0.856 |
| BMI | 0.011 | <0.001 | 0.007 |
| HR | 0.031 | 0.541 | 0.263 |
| **Laboratory tests** |  |  |  |
| Hgb | 0.880 | <0.001 | <0.001 |
| Platelets | 0.028 | 0.680 | 0.416 |
| WBC | 0.252 | <0.001 | <0.001 |
| Ca | <0.001 | <0.001 | 0.002 |
| Na | 0.945 | <0.001 | <0.001 |
| Cl | <0.001 | <0.001 | <0.001 |
| K | <0.001 | <0.001 | <0.001 |
| BG | 0.987 | <0.001 | <0.001 |
| Cr | <0.001 | <0.001 | <0.001 |
| BUN | <0.001 | <0.001 | <0.001 |
| **Comorbidity diseases** |  |  |  |
| Myocardial infarct | 0.004 | <0.001 | 0.364 |
| Congestive heart failure | 0.285 | <0.001 | 0.001 |
| Cerebrovascular disease | 0.046 | <0.001 | 0.014 |
| Chronic pulmonary disease | 1.000 | <0.001 | <0.001 |
| Respiratory failure | 0.001 | 0.301 | <0.001 |
| Liver disease | 0.004 | 0.143 | <0.001 |
| Kidney disease | <0.001 | <0.001 | <0.001 |
| Malignant cancer | 0.901 | 0.019 | 0.237 |
| Diabetes | <0.001 | <0.001 | <0.001 |
| Infection | 0.005 | 0.130 | 0.754 |
| **AKI stage** | 1.000 | <0.001 | <0.001 |
| **Severity of illness** |  |  |  |
| SOFA score | 0.716 | <0.001 | <0.001 |
| Comorbidity index | 0.007 | <0.001 | <0.001 |
| Saps II | 0.227 | <0.001 | <0.001 |
| **Interventions (day 1)** |  |  |  |
| Mechanical ventilation | 0.045 | 0.034 | 1.000 |
| Diuretics use | <0.001 | 0.037 | 0.298 |
| Vasoactive drugs use | 0.095 | <0.001 | 0.045 |
| RRT use | 1.000 | <0.001 | <0.001 |
| **Outcomes** |  |  |  |
| ICU 30-day mortality | 0.062 | 0.018 | <0.001 |

BMI, body mass index; HR, heart rate; Hgb, hemoglobin; WBC, white blood cell; Ca, calcium; Na, sodium; Cl, chlorine; K, potassium; BG, blood glucose; Cr, creatinine; BUN, blood urea nitrogen; SOFA, sequential organ failure assessment; Saps II, simplified acute physiology score II; RRT, renal replacement therapy.
