# Supplementary material for: Association between admission baseline blood potassium levels and all-cause mortality in patients with acute kidney injury combined with sepsis: A retrospective cohort study
Source: PLoS One. 2024 Nov 20;19(11):e0309764. doi: 10.1371/journal.pone.0309764 (PMC11578480; doi:10.1371/journal.pone.0309764)
Supplement: S2 Table — ✝: The continuous variables goes up per 10 units. ↗: The categorical variables are referenced by ‘No’. BMI, body mass index; HR, heart rate; RR, respiration rate; SBP, systolic blood pressure; DBP, diastolic blood pressure; Hgb, hemoglobin; WBC, white blood cell; Ca, calcium; Na, sodium; Cl, chlorine; K, potassium; BG, blood glucose; Cr, creatinine; BUN, blood urea nitrogen; SOFA, sequential organ failure assessment; Saps II, simplified acute physiology score II; RRT, renal replacement therapy. (DOCX) [file pone.0309764.s002.docx]

**S2 Table. Results of univariate analysis of ICU 30-day mortality.**

| **Variables** | **ICU 30-day mortality** | |
| --- | --- | --- |
|  | **HR (95%CI)** | ***p*-value** |
| Sex (male vs. female) | 1.06 (0.94,1.19) | 0.375 |
| Age (years) | 1.02 (1.01,1.02) | < 0.001 |
| BMI (kg/m^2^) | 0.98 (0.98,0.99) | < 0.001 |
| HR (bmp)✝ | 1.05 (1.02,1.07) | < 0.001 |
| RR (bmp) | 1.02 (1.01,1.03) | < 0.001 |
| SBP (mmHg)✝ | 0.94 (0.92,0.97) | < 0.001 |
| DBP (mmHg)✝ | 0.99 (0.96,1.02) | 0.362 |
| **Laboratory tests** |  |  |
| Hgb (g/dL) | 0.95 (0.93,0.98) | < 0.001 |
| Platelets (×10^9/L)✝ | 0.99 (0.98,1.00) | 0.026 |
| WBC (×10^9/L)✝ | 1.07 (1.04,1.09) | < 0.001 |
| Ca (mmol/L) | 0.91 (0.86,0.97) | 0.003 |
| Na (mmol/L) | 0.98 (0.97,0.99) | < 0.001 |
| Cl (mmol/L) | 0.97 (0.96,0.98) | < 0.001 |
| K (mmol/L) | 1.20 (1.12,1.28) | < 0.001 |
| BG (mg/dL)✝ | 1.01 (1.00,1.02) | < 0.001 |
| Cr (mg/dl) | 1.09 (1.06,1.12) | < 0.001 |
| BUN (mg/dl) | 1.01 (1.01,1.01) | < 0.001 |
| **Comorbidity diseases** |  |  |
| Myocardial infarct↗ | 1.63 (1.42,1.86) | < 0.001 |
| Congestive heart failure↗ | 1.30 (1.15,1.46) | < 0.001 |
| Cerebrovascular disease↗ | 0.92 (0.79,1.07) | 0.298 |
| Chronic pulmonary disease↗ | 1.13 (0.99,1.28) | 0.06 |
| Respiratory failure↗ | 1.38 (1.21,1.59) | < 0.001 |
| Liver disease↗ | 1.61 (1.41,1.83) | < 0.001 |
| Kidney disease↗ | 1.22 (1.07,1.40) | 0.003 |
| Malignant cancer↗ | 1.37 (1.16,1.61) | < 0.001 |
| Diabetes↗ | 0.89 (0.78,1.01) | 0.079 |
| Infection↗ | 0.50 (0.44,0.57) | < 0.001 |
| **AKI stage** |  | < 0.001 |
| 1 | ref |  |
| 2 | 1.01 (0.84,1.21) | 0.943 |
| 3 | 2.00 (1.68,2.38) | < 0.001 |
| **Severity of illness** |  |  |
| SOFA score | 1.07 (1.05,1.10) | < 0.001 |
| Comorbidity index | 1.12 (1.10,1.14) | < 0.001 |
| Saps II | 1.03 (1.03,1.04) | < 0.001 |
| **Interventions (day 1)** |  |  |
| Mechanical ventilation↗ | 1.35 (0.97,1.88) | 0.076 |
| Diuretics use↗ | 0.32 (0.28,0.36) | < 0.001 |
| Vasoactive drugs use↗ | 2.32 (1.94,2.78) | < 0.001 |
| RRT use↗ | 1.63 (1.43,1.86) | < 0.001 |
| **K (mmol/L)** |  | < 0.001 |
| T1 < 3.9 | 1.06 (0.91,1.24) | 0.467 |
| 3.9 ≤ T2 < 4.5 | ref |  |
| T3 ≥ 4.5 | 1.38 (1.20,1.59) | < 0.001 |

✝: The continuous variables goes up per 10 units.

↗: The categorical variables are referenced by ‘No’.

BMI, body mass index; HR, heart rate; RR, respiration rate; SBP, systolic blood pressure; DBP, diastolic blood pressure; Hgb, hemoglobin; WBC, white blood cell; Ca, calcium; Na, sodium; Cl, chlorine; K, potassium; BG, blood glucose; Cr, creatinine; BUN, blood urea nitrogen; SOFA, sequential organ failure assessment; Saps II, simplified acute physiology score II; RRT, renal replacement therapy.
