# Supplementary material for: Association between admission baseline blood potassium levels and all-cause mortality in patients with acute kidney injury combined with sepsis: A retrospective cohort study
Source: PLoS One. 2024 Nov 20;19(11):e0309764. doi: 10.1371/journal.pone.0309764 (PMC11578480; doi:10.1371/journal.pone.0309764)
Supplement: S4 Table — Hazard ratios (HRs) were adjusted for age, sex, BMI, Hgb, BG, Cr, myocardial infarct, congestive heart failure, respiratory failure, kidney disease, malignant cancer, SOFA score, comorbidity index. BMI, body mass index; Hgb, hemoglobin; BG, blood glucose; Cr, creatinine; SOFA, sequential organ failure assessment. (DOCX) [file pone.0309764.s004.docx]

**S4 Table. Interactive effect of blood K levels and ICU 30-day mortality in patients with and without RRT use.**

| **Variables** | **without RRT (n=7,320)** | | **with RRT (n=922)** | | ***p*-value for the interaction** |
| --- | --- | --- | --- | --- | --- |
|  | **HR (95%CI)** | ***p*-value** | **HR (95%CI)** | ***p*-value** |  |
| K (continuous) | 1.12 (1.04~1.20) | 0.003 | 1.12 (1.00~1.27) | 0.056 | 0.168 |
| K (tertiles) |  |  |  |  |  |
| T1 (< 3.9) | 1.02 (0.88~1.17) | 0.801 | 1.21 (0.86~1.72) | 0.274 | 0.118 |
| T2 (3.9~4.5) | ref |  | ref |  |  |
| T3 (≥ 4.5) | 1.13 (0.99~1.30) | 0.080 | 1.46 (1.09~1.96) | 0.012 |  |
| trend test |  | 0.176 |  | 0.373 |  |

Hazard ratios (HRs) were adjusted for age, sex, BMI, Hgb, BG, Cr, myocardial infarct, congestive heart failure, respiratory failure, kidney disease, malignant cancer, SOFA score, comorbidity index. BMI, body mass index; Hgb, hemoglobin; BG, blood glucose; Cr, creatinine; SOFA, sequential organ failure assessment.
