# Supplementary material for: Association between admission baseline blood potassium levels and all-cause mortality in patients with acute kidney injury combined with sepsis: A retrospective cohort study
Source: PLoS One. 2024 Nov 20;19(11):e0309764. doi: 10.1371/journal.pone.0309764 (PMC11578480; doi:10.1371/journal.pone.0309764)
Supplement: S6 Table — Model 1 adjust for age and sex. Model 2 adjust for Model 1 + BMI, Hgb, BG, Cr. Model 3 adjust for Model 1 + Model 2 + myocardial infarct, congestive heart failure, respiratory failure, kidney disease, malignant cancer, SOFA score, comorbidity index. BMI, body mass index; Hgb, hemoglobin; BG, blood glucose; Cr, creatinine; SOFA, sequential organ failure assessment. (DOCX) [file pone.0309764.s006.docx]

**S6 Table. Multivariable-adjust HRs and 95%CI of blood K levels associated with ICU 30-day mortality (grouped according to clinical cut-off values).**

| **Variables** | **Unadjusted** | | **Model 1** | | **Model 2** | | **Model 3** | |
| --- | --- | --- | --- | --- | --- | --- | --- | --- |
|  | **HR (95%CI)** | ***p*-value** | **HR (95%CI)** | ***p*-value** | **HR (95%CI)** | ***p*-value** | **HR (95%CI)** | ***p*-value** |
| K (continuous) | 1.21 (1.16~1.27) | <0.001 | 1.22 (1.16~1.28) | <0.001 | 1.14 (1.07~1.20) | <0.001 | 1.12 (1.03~1.22) | 0.006 |
| K (tertiles) |  |  |  |  |  |  |  |  |
| T1 (< 3.5) | 1.04 (0.93~1.15) | 0.490 | 1.06 (0.95~1.17) | 0.310 | 1.08 (0.95~1.22) | 0.246 | 1.19 (1.00~1.41) | 0.047 |
| T2 (3.5~5.5) | ref |  | ref |  | ref |  | ref |  |
| T3 (≥5.5) | 1.33 (1.20~1.46) | <0.001 | 1.34 (1.21~1.47) | <0.001 | 1.24 (1.11~1.40) | <0.001 | 1.28 (1.09~1.50) | 0.003 |
| *P* for trend |  | <0.001 |  | <0.001 |  | <0.001 |  | 0.008 |

Model 1 adjust for age and sex.

Model 2 adjust for Model 1 + BMI, Hgb, BG, Cr.

Model 3 adjust for Model 1 + Model 2 + myocardial infarct, congestive heart failure, respiratory failure, kidney disease, malignant cancer, SOFA score, comorbidity index.

BMI, body mass index; Hgb, hemoglobin; BG, blood glucose; Cr, creatinine; SOFA, sequential organ failure assessment.
