# Supplementary material for: Association between admission baseline blood potassium levels and all-cause mortality in patients with acute kidney injury combined with sepsis: A retrospective cohort study
Source: PLoS One. 2024 Nov 20;19(11):e0309764. doi: 10.1371/journal.pone.0309764 (PMC11578480; doi:10.1371/journal.pone.0309764)
Supplement: S7 Table — Model 1 adjust for age and sex. Model 2 adjust for Model 1 + BMI, Hgb, BG, Cr. Model 3 adjust for Model 1 + Model 2 + myocardial infarct, congestive heart failure, respiratory failure, kidney disease, malignant cancer, SOFA score, comorbidity index. BMI, body mass index; Hgb, hemoglobin; BG, blood glucose; Cr, creatinine; SOFA, sequential organ failure assessment. (DOCX) [file pone.0309764.s007.docx]

**S7 Table. Multivariable-adjust HRs and 95%CI of blood K levels associated with ICU 30-day mortality (before excluding missing values).**

| **Variables** | **Unadjusted** | | **Model 1** | | **Model 2** | | **Model 3** | |
| --- | --- | --- | --- | --- | --- | --- | --- | --- |
|  | **HR (95%CI)** | ***p*-value** | **HR (95%CI)** | ***p*-value** | **HR (95%CI)** | ***p*-value** | **HR (95%CI)** | ***p*-value** |
| K (continuous) | 1.23 (1.16~1.30) | <0.001 | 1.25 (1.18~1.32) | <0.001 | 1.15 (1.07~1.23) | <0.001 | 1.12 (1.05~1.20) | 0.001 |
| K (tertiles) |  |  |  |  |  |  |  |  |
| T1 (< 3.9) | 1.06 (0.93~1.20) | 0.390 | 1.07 (0.94~1.22) | 0.291 | 1.09 (0.94~1.27) | 0.264 | 1.07 (0.92~1.25) | 0.370 |
| T2 (3.9~4.5) | ref |  | ref |  | ref |  | ref |  |
| T3 (≥4.5) | 1.37 (1.22~1.54) | <0.001 | 1.39 (1.24~1.56) | <0.001 | 1.29 (1.11~1.48) | 0.001 | 1.24 (1.07~1.43) | 0.004 |
| *P* for trend |  | <0.001 |  | <0.001 |  | 0.018 |  | 0.039 |

Model 1 adjust for age and sex.

Model 2 adjust for Model 1 + BMI, Hgb, BG, Cr.

Model 3 adjust for Model 1 + Model 2 + myocardial infarct, congestive heart failure, respiratory failure, kidney disease, malignant cancer, SOFA score, comorbidity index.

BMI, body mass index; Hgb, hemoglobin; BG, blood glucose; Cr, creatinine; SOFA, sequential organ failure assessment.
