# Supplementary material for: Association between admission baseline blood potassium levels and all-cause mortality in patients with acute kidney injury combined with sepsis: A retrospective cohort study
Source: PLoS One. 2024 Nov 20;19(11):e0309764. doi: 10.1371/journal.pone.0309764 (PMC11578480; doi:10.1371/journal.pone.0309764)
Supplement: S1 Fig — (DOCX) [file pone.0309764.s008.docx]

**
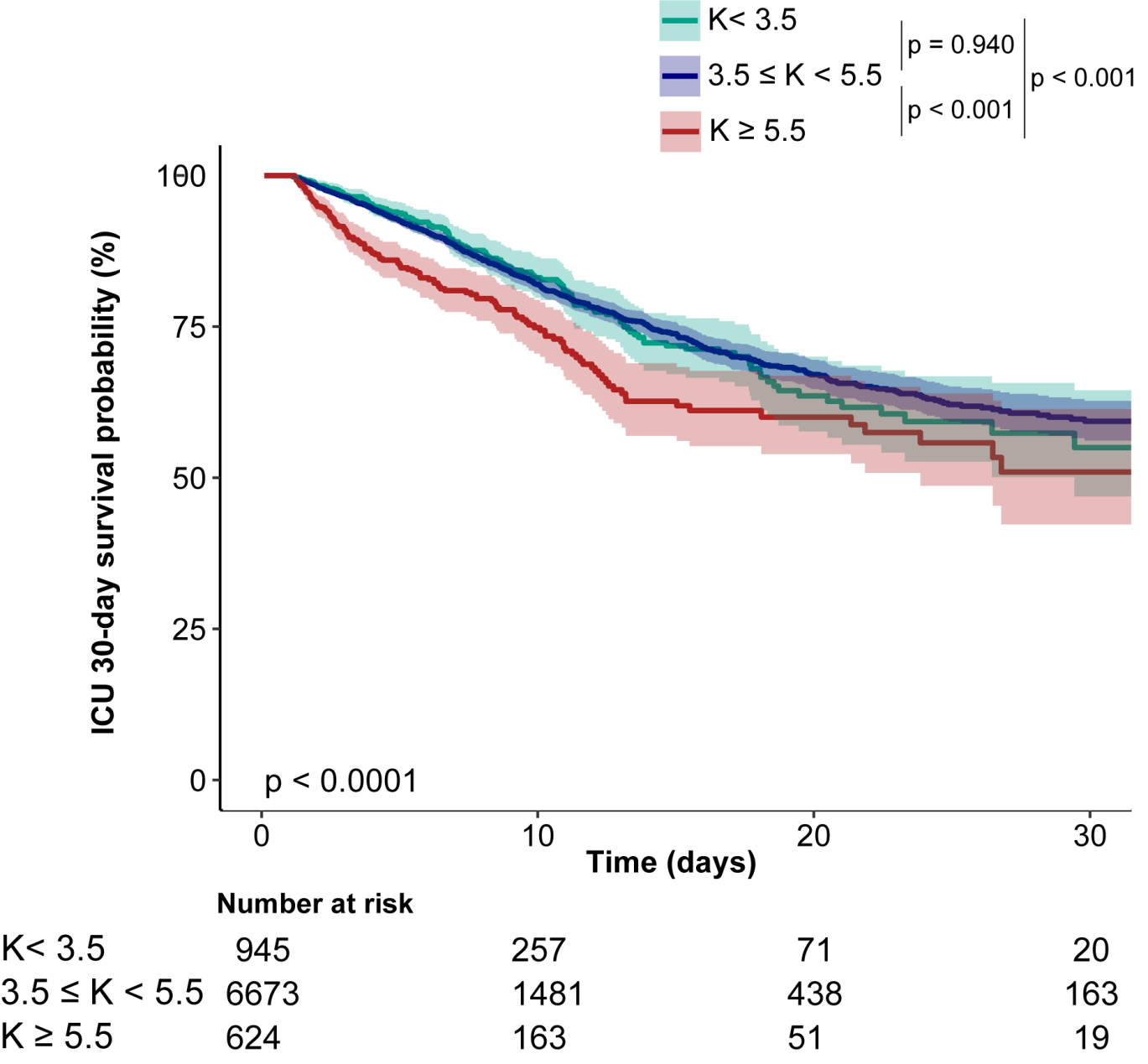
**

**S1 Fig. Kaplan–Meier survival curves for ICU 30-day mortality (grouped according to clinical cut-off values).**
